# Relationships of adiponectin to regional adiposity, insulin sensitivity, serum lipids, and inflammatory markers in sedentary and endurance-trained Japanese young women

**DOI:** 10.3389/fendo.2023.1097034

**Published:** 2023-01-24

**Authors:** Yaxin Guan, Fan Zuo, Juan Zhao, Xin Nian, Li Shi, Yushan Xu, Jingshan Huang, Tsutomu Kazumi, Bin Wu

**Affiliations:** ^1^ Department of Endocrinology, First Affiliated Hospital of Kunming Medical University, Kunming, Yunnan, China; ^2^ Yunnan Province Clinical Medical Center for Endocrine and Metabolic Diseases, Kunming, Yunnan, China; ^3^ School of Computing, University of South Alabama, Mobile, AL, United States; ^4^ Open Research Center for Studying of Lifestyle-Related Diseases, Mukogawa Women’s University, Nishinomiya, Japan; ^5^ Department of Food Sciences and Nutrition, School of Human Environmental Science, Mukogawa Women’s University, Nishinomiya, Japan; ^6^ Research Institute for Nutrition Sciences, Mukogawa Women’s University, Nishinomiya, Japan

**Keywords:** adiponectin, physical activity, regional adiposity, insulin resistance, diabetes

## Abstract

**Introduction:**

This study aims to compare the differences in circulating adiponectin levels and their relationships to regional adiposity, insulin resistance, serum lipid, and inflammatory factors in young, healthy Japanese women with different physical activity statuses.

**Methods:**

Adipokines (adiponectin and leptin), full serum lipid, and inflammatory factors [white blood cell counts, C-reactive protein, tumor necrosis factor-α, tissue plasminogen activator inhibitor-1 (PAI-1)] were measured in 101 sedentary and 100 endurance-trained healthy Japanese women (aged 18–23 years). Insulin sensitivity was obtained through a quantitative insulin-sensitivity check index (QUICKI). Regional adiposity [trunk fat mass (TFM), lower-body fat mass (LFM), and arm fat mass (AFM)] was evaluated using the dual-energy X-ray absorptiometry method.

**Results:**

No significant difference was observed between the sedentary and trained women in terms of adiponectin levels. The LFM-to-TFM ratio and the high-density lipoprotein cholesterol (HDL-C) were the strong positive determinants for adiponectin in both groups. Triglyceride in the sedentary women was closely and negatively associated with adiponectin, as well as PAI-1 in the trained women. The QUICKI level was higher in the trained than sedentary women. However, no significant correlation between adiponectin and insulin sensitivity was detected in both groups. Furthermore, LFM was associated with a favorable lipid profile against cardiovascular diseases (CVDs) in the whole study cohort, but this association became insignificant when adiponectin was taken into account.

**Conclusions:**

These findings suggest that adiponectin is primarily associated with regional adiposity and HDL-C regardless of insulin sensitivity and physical activity status in young, healthy women. The associations among adiponectin, lipid, and inflammatory factors are likely different in women with different physical activity statuses. The correlation of LFM and a favorable lipid profile against CVD and adiponectin is likely involved in this association.

## 1 Introduction

Energy over intaking and physical inactivity are two major risk factors for the development of obesity, type 2 diabetes, and many aspects of metabolic syndrome, which are attributed to insulin resistance (1). Moderate physical activity is currently recommended for obese or overweight individuals to reduce the risk of type 2 diabetes and metabolic syndrome (2). However, the mechanisms through which physical activity improves insulin sensitivity remain unclear. A single bout of exercise intervention in both diabetic and non-diabetic individuals can acutely improve insulin sensitivity, but this effect dissipates within days (3, 4). Long-term exercise interventions cannot effectively improve insulin activity without weight improvement (5, 6). These findings indicate that the effect of physical activity on insulin sensitivity is partly mediated by the reduction of body weight and/or body fat mass. One of the main effects of physical activity on body mass distribution is the prevention of subcutaneous fat mass from transferring into the abdominal cavity and leading to a major deposition of adipose tissue in the subcutaneous region (7, 8).

The lower-body region is one of the major areas for the accumulation of subcutaneous adipose tissue. Different adipose depositions have been recognized to cause different metabolic consequences (9, 10). Those who accumulate fat tissue in the trunk region (android obese) are more likely to develop diabetes and cardiovascular diseases (CVDs) than those with major lower-body-fat mass (LFM) deposition (gynoid obese) (10). One of the reasons for the higher prevalence of CVDs in men than in women is that men tend to develop android obesity, whereas women tend to develop gynoid obesity (11). LFM has been reported to play a protective role for CVDs due to its association with a favorable serum lipid profile and increased insulin sensitivity (11). However, the mediator of the interaction between body fat mass distribution and insulin sensitivity, as well as lipid metabolism, warrants further investigation.

Adiponectin is a peptide expressed specifically and abundantly in adipose tissue (12, 13) and has been suggested to be an important regulator of insulin action, thereby possibly linking adiposity with insulin sensitivity (14). Circulating adiponectin levels are reduced in individuals with obesity (15) and diabetes (16). A longitudinal study in Pima Indians presented that a high concentration of plasma adiponectin strongly predicts a lower incidence rate of type 2 diabetes independent of obesity (17). Furthermore, low adiponectin concentrations have been associated with a higher risk of type 2 diabetes (17, 18) and a more atherogenic lipid profile (19).

To date, the relationships among adiponectin, physical activity, and body fat mass distribution are equivocal. A study indicated that moderate physical activity training might improve adiponectin levels in middle-aged adults predisposed to metabolic syndrome (20). Another study including eight healthy subjects showed that circulating adiponectin concentration was increased by physical exercise training when body fat content was reduced but did not change when the body composition was unaltered (21). A study involving 40 obese young women demonstrated that no changes were observed in adiponectin levels after a nine-week intervention (22). In the present research, we conducted a cross-sectional study involving 101 sedentary and 100 endurance-trained healthy Japanese young women to investigate the circulating adiponectin levels and their relationships with regional adiposity, insulin sensitivity, serum lipid, and inflammatory markers. We aimed to explore the potential links between adiponectin with regional adiposity and various metabolic parameters in women with different physical activity statuses. Moreover, we tested the association of adiponectin with the insulin-sensitizing effects of physical activity and LFM.

## 2 Materials and methods

### 2.1 Study participants

The study population comprised 201 young women (aged 18–23 years) who are students of Mukogawa Women’s University (MWU) in Nishinomiya, Japan. The study was approved by the MWU ethics committee, and written informed consent was obtained from each participant. The selection and recruitment procedures were described previously (23). The subjects in this study were categorized into two groups according to their physical activity habits. The 101 sedentary untrained students recruited from the Department of Food Sciences and Nutrition were not engaged in any regular sport activity. The 100 endurance-trained athletes were recruited from members of a volleyball club (28 students), a basketball club (46 students), and a track club (26 students). They have been training five hours per day and 5–7 days a week for two years or longer and participate regularly in competitive events in their respective sports specialties. All of them had similar anthropometric indices. All but six women were nonsmokers, and none had recently been on a diet or consumed alcohol daily. Neither did any of them receive medications.

### 2.2 Anthropometric and regional fat mass distribution

**Figure 1:**
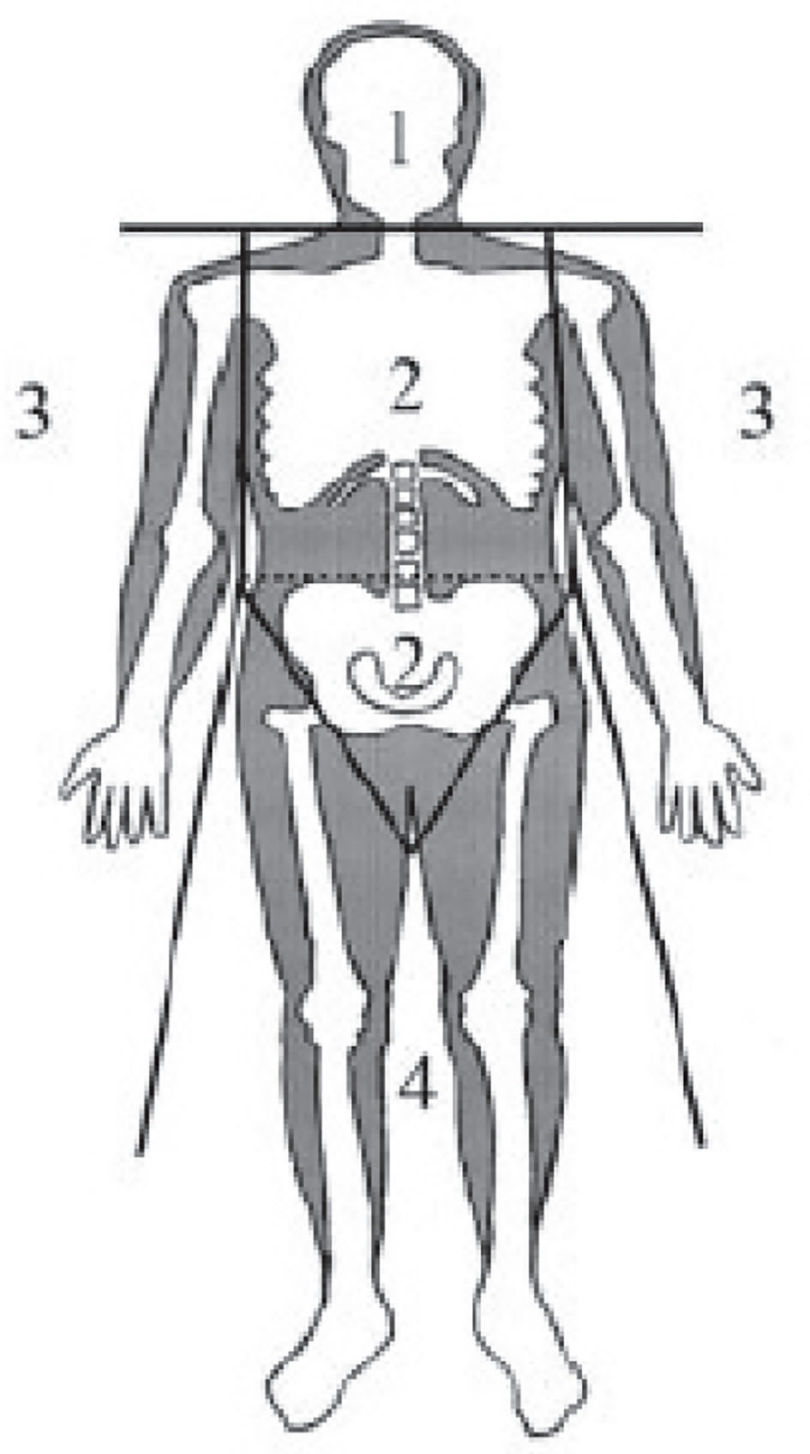
Standard regions of a dual-energy X-ray absorptiometry scan: 1, head; 2, trunk; 3, arms; 4, lower body.

Body mass index (BMI) was calculated as weight (kg)/[height (m)]^2^. A dual-energy X-ray absorptiometry with a scanner (Hologic QDR-2000, Waltham, MA) was applied to measure regional fat mass distribution. A scanned image of the whole body was divided into six subdivisions: head, trunk, left and right arms, and left and right limbs. The dividing borders between these subregions were differentiated by a line underneath the chin, a line between the humerus head and the glenoid fossa, and a line at the femoral neck (**Figure 1**). Trunk fat mass (TFM), also known as android fat mass, has been documented to be strongly and positively related to visceral adiposity measured with magnetic resonance imaging (24). The following parameters were introduced to describe regional fat deposition: i) total body fat mass ratio (% total fat), illustrated as a percentage of total fat tissue weight/body weight; ii) LFM ratio (L/Tr ratio), illustrated as LFM/TFM; and iii) arm fat ratio (A/Tr), illustrated as arm fat mass/TFM.

### 2.3 Glucose, insulin, and insulin resistance

Plasma glucose was measured through the hexokinase method [interassay coefficiency of variation (CV) < 2%]. Insulin was measured by an enzyme-linked immunosorbent assay (ELISA) with narrow specificity, excluding des-31, des-32, and intact proinsulin (Abbott Japan, Tokyo, Japan, interassay CV = 3.3%). The quantitative insulin-sensitivity check index (QUICKI) was used as a surrogate index for insulin sensitivity. QUICKI has an excellent linear correlation with the glucose clamp index of insulin sensitivity and is regarded as one of the most accurate surrogate indexes to determine human insulin sensitivity (25). QUICKI was calculated using the following formula: QUICKI = 1/[log(I_0_) + log(G_0_)], where I_0_ is the fasting insulin (microunits per milliliter) and G_0_ is the fasting glucose (milligrams per deciliter).

### 2.4 Lipids, lipoprotein, and apolipoprotein

Serum lipids [triglycerides (TG), total cholesterol (TC), high-density lipoprotein cholesterol (HDL-C)] were measured using an autoanalyzer (AU5232, Olympus, Tokyo, Japan). Apolipoprotein A-1 (ApoA1) and apolipoprotein B-100 (ApoB) were measured with respective commercial kits using an Olympus autoanalyzer (AU600, Mitsubishi Chemicals, Tokyo, Japan). Low-density lipoprotein cholesterol (LDL-C) was determined using the Friedewald formula. The interassay CV were as follows: 5.0% for TG, 1.1% for TC, 3% for HDL-C, 5.0% for ApoA1, and 2.0% for ApoB.

### 2.5 Adipokines

Adiponectin was assayed by a sandwich ELISA employing an adiponectin-specific antibody. The intra- and inter-assay CV were 3.3% and 7.5%, respectively (Otsuka Pharmaceutical Co., Ltd., Tokushima City, Japan). Leptin was assessed by a radioimmunoassay kit purchased from LINCO Research (St. Charles, MO, interassay CV = 4.9%).

### 2.6 Inflammatory and acute response markers

White blood cell counts (WBC) were measured by an XE-2100 automatic blood routine analyzer (Sysmex Corporation, Kobe, Japan). Serum highly sensitive C-reactive protein (hsCRP) concentration was measured by an immunoturbidometric assay with reagents and calibrators purchased from Dade Behring Marbura GmbH (Marburg, Germany; inter-assay CV < 5.0%). Tumor necrosis factor-α (TNF-α) was measured by immunoassays (R&D Systems, Inc., Minneapolis, MN, interassay CV = 6.0%). Tissue plasminogen activator inhibitor-1 (PAI-1) was measured by an ELISA method (Mitsubishi Chemicals, interassay CV = 8.1%).

### 2.7 Statistical analysis

Data were expressed as mean ± SD. The normality of data distribution was examined using the Kolmogorov-Smirnov test. Comparison of demographic and metabolic variables was carried out by unpaired t-test and Mann-Whitney U test when the data were distributed non-normally. Correlations were conducted by univariate linear regression. Partial correlation analysis was applied to assess the relationship between two variables when confounding factors need to be adjusted. Multiple regression analysis was used to determine whether the association between the dependent and independent variables of interest remained significant after adjusting for other potentially confounding independent variables. The stepwise regression model was used to estimate the relative contribution of the independent variables and the variability of the dependent variable. Data were considered statistically significant when the p-value ≤ 0.05. All statistical calculations were performed using SPSS 27.0 (Chicago, IL).

## 3 Results

### 3.1 Anthropometric, regional adiposity, and metabolic characteristics

The two groups were matched by age. For body fat mass distribution, compared with the endurance-trained women, the sedentary subjects had higher total fat mass (+1.8 kg, p = 0.002), % total fat (+6.6%, p < 0.001), TFM (+1.0 kg, p = 0.003), % TFM (+3.1%, p < 0.001), arm FM (+0.21 kg, p = 0.006), and LFM (+0.48 kg, p = 0.018). Meanwhile, the BMI value was slightly lower in the sedentary women (–0.9 kg/m^2^, p = 0.003). The A/Tr and L/Tr ratios were similar between the two groups. Systolic blood pressure (SBP) was slightly higher in the endurance-trained subjects (+3 mmHg, p = 0.008), while diastolic blood pressure (DBP) was similar between the two groups. For adipokines, a higher leptin concentration was observed in the sedentary group (+2.86 ng/ml, p < 0.001), although the adiponectin level was comparable between groups (10.77 ± 3.70 μg/ml in sedentary *vs.* 10.96 ± 4.12 μg/ml in trained, p = 0.743). For insulin sensitivity, QUICKI was lower in the sedentary subjects (–0.02U, p = 0.002). For the lipid profiles, LDL-C, ApoB, and ApoB/ApoA1 were higher in the sedentary subjects. For inflammatory factors, WBC, TNF-α, and hsCRP were similar between groups (**Table 1**).

**Table 1:** Anthropometric, regional adiposity, and metabolic characteristics (X ± SD).

	Sedentary	Endurance-Trained	P-Value
n	101	100	*NA*
Age (years)	20.3 ± 1.2	19.3 ± 1.2	0.278
BMI (kg/m^2^)	20.61 ± 2.17	21.51 ± 2.00	**0.003**
Total Fat Mass (kg)	15.32 ± 4.39	13.51 ± 3.90	**0.002**
% Total Fat	29.4 ± 5.2	22.8 ± 4.7	**<0.001**
TFM (kg)	7.41 ± 2.58	6.41 ± 2.10	**0.003**
%TFM	14.0 ± 3.3	10.9 ± 3.0	**<0.001**
AFM (kg)	1.37 ± 0.56	1.16 ± 0.54	**0.006**
A/Tr (%)	19.0 ± 5.0	18.0 ± 6.0	0.271
LFM (kg)	5.91 ± 1.44	5.43 ± 1.42	**0.018**
L/Tr (%)	83.5 ± 15.3	87.6 ± 14.7	0.052
SBP (mmHg)	103.3 ± 7.3	106.3 ± 8.5	**0.008**
DBP (mmHg)	57.1 ± 4.9	56.4 ± 6.1	0.399
Adiponectin (μg/ml)	10.77 ± 3.7	10.96 ± 4.12	0.743
Leptin (ng/ml)	9.36 ± 3.99	6.50 ± 2.63	**<0.001**
FPG (mmol/L)	4.76 ± 0.38	4.78 ± 0.39	0.441
Fasting insulin (μU/ml)	7.48 ± 4.97	5.15 ± 2.73	**<0.001**
QUICKI	0.37 ± 0.04	0.39 ± 0.04	**0.002**
TG (mmol/L)	0.66 ± 0.27	0.62 ± 0.26	0.324
TC (mmol/L)	4.69 ± 0.68	4.54 ± 0.66	0.105
HDL-C (mmol/L)	1.96 ± 0.35	2.00 ± 0.36	0.353
LDL-C (mmol/L)	2.43 ± 0.59	2.25 ± 0.52	**0.020**
ApoA1 (mg/dl)	164.23 ± 20.84	169.97 ± 21.93	0.058
ApoB (mg/dl)	73.41 ± 14.36	68.97 ± 12.96	**0.023**
ApoB/ApoA1	0.46 ± 0.12	0.41 ± 0.09	**0.004**
PAI-1 (ng/ml)	17.64 ± 8.86	16.74 ± 7.40	0.437
WBC (/μl)	6122 ± 1698	5728 ± 1469	0.081
TNF-α (pg/ml)	0.58 ± 0.64	0.50 ± 0.37	0.299
Log (hsCRP)	1.00 ± 0.48	1.02 ± 0.47	0.750

Numbers in bold: with statistical significance.

### 3.2 Univariate correlations

#### 3.2.1 Association of adiponectin with body fat mass distribution

**Figure 2:**
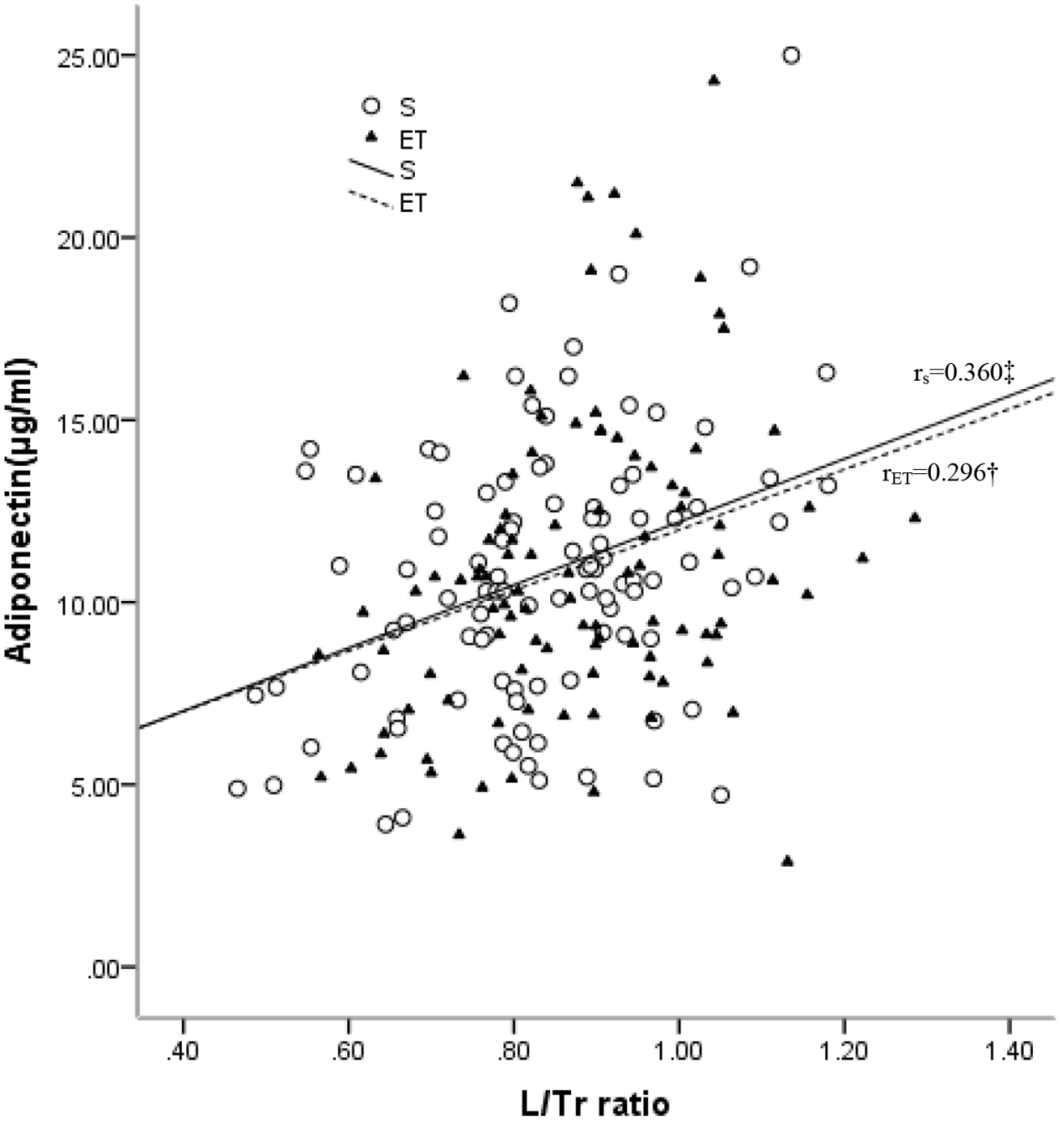
Relationships between plasma levels of adiponectin and L/Tr ratio in the sedentary (S) and endurance-trained (ET) groups. r_S_: correlation coefficient of the sedentary group; r_ET:_ correlation coefficient of the endurance-trained group. *: P < 0.05; †: P < 0.01; ‡: P < 0.001 .

A simple correlation analysis (**Table 2**) revealed that adiponectin was reversely associated with BMI (r = –0.211, p = 0.034), TFM (r = –0.221, p = 0.027), and % TFM (r = –0.197, p = 0.048) and positively associated with L/Tr (r = 0.360, p < 0.001, **Figure 2**) in the sedentary individuals. For the endurance-trained women, only the L/Tr ratio was found to have a positive association with adiponectin (r = 0.296, p = 0.003, **Figure 2**). Notably, adiponectin was positively associated with total fat mass (r = 0.315, p < 0.001), % total fat (r = 0.241, p = 0.016), LFM (r = 0.292, p = 0.003), and L/Tr ratio (r = 0.291, p = 0.003) after adjustment for TFM in the sedentary group. For the endurance-trained women, plasma adiponectin was positively associated with total fat mass (r = 0.400, p<0.001), LFM (r = 0.393, p < 0.001), and L/Tr ratio (r = 0.331, p = 0.001) after adjustment for TFM (**Table 2**).

**Table 2:** Correlations of adiponectin with regional adiposity and metabolic variables in the two groups before and after adjustment for TFM.

	Sedentary	Endurance-Trained	Sedentary	Endurance-Trained
	Not adjusted		Adjusted for TFM	
	r	r	r	r
BMI	-0.211^*^	0.064	–0.040	0.150
Total Fat Mass	-0.142	0.041	0.315**^‡^**	0.400**^‡^**
%Total fat	-0.093	0.035	0.241^*^	0.206^*^
TFM	-0.221^*^	-0.053	NA	NA
% TFM	-0.197^*^	-0.068	0.042	–0.056
AFM	0.073	0.082	0.166	0.185
A/Tr	0.144	0.166	0.132	0.176
LFM	-0.003	0.159	0.292**^†^**	0.393**^‡^**
L/Tr ratio	0.360**^‡^**	0.296**^†^**	0.291**^†^**	0.331**^‡^**
Leptin	-0.089	-0.056	0.025	–0.062
FPG	0.079	-0.085	0.070	–0.094
QUICKI	0.032	-0.024	–0.031	–0.036
TG	-0.297**^†^**	-0.089	–0.258**^†^**	–0.092
TC	0.209	0.177	0.271**^†^**	0.176
HDL-C	0.431**^‡^**	0.241^*^	0.420**^‡^**	0.237^*^
LDL-C	0.047	0.082	0.112	0.086
ApoA1	0.373**^‡^**	0.223^*^	0.363**^‡^**	0.218^*^
ApoB	-0.087	0.021	–0.007	0.024
ApoB/ApoA1	-0.253^*^	-0.122	–0.194	–0.116
PAI-1	-0.162	-0.216^*^	–0.088	–0.210^*^
WBC	-0.192	-0.070	–0.157	–0.068
TNF-α	0.011	-0.160	–0.004	–0.160
Log (hsCRP)	-0.196^*^	-0.127	–0.163	–0.156

*: P < 0.05; †: P < 0.01; ‡: P < 0.001; NA, not applicable.

#### 3.2.2 Association of adiponectin with serum lipids

**Figure 3:**
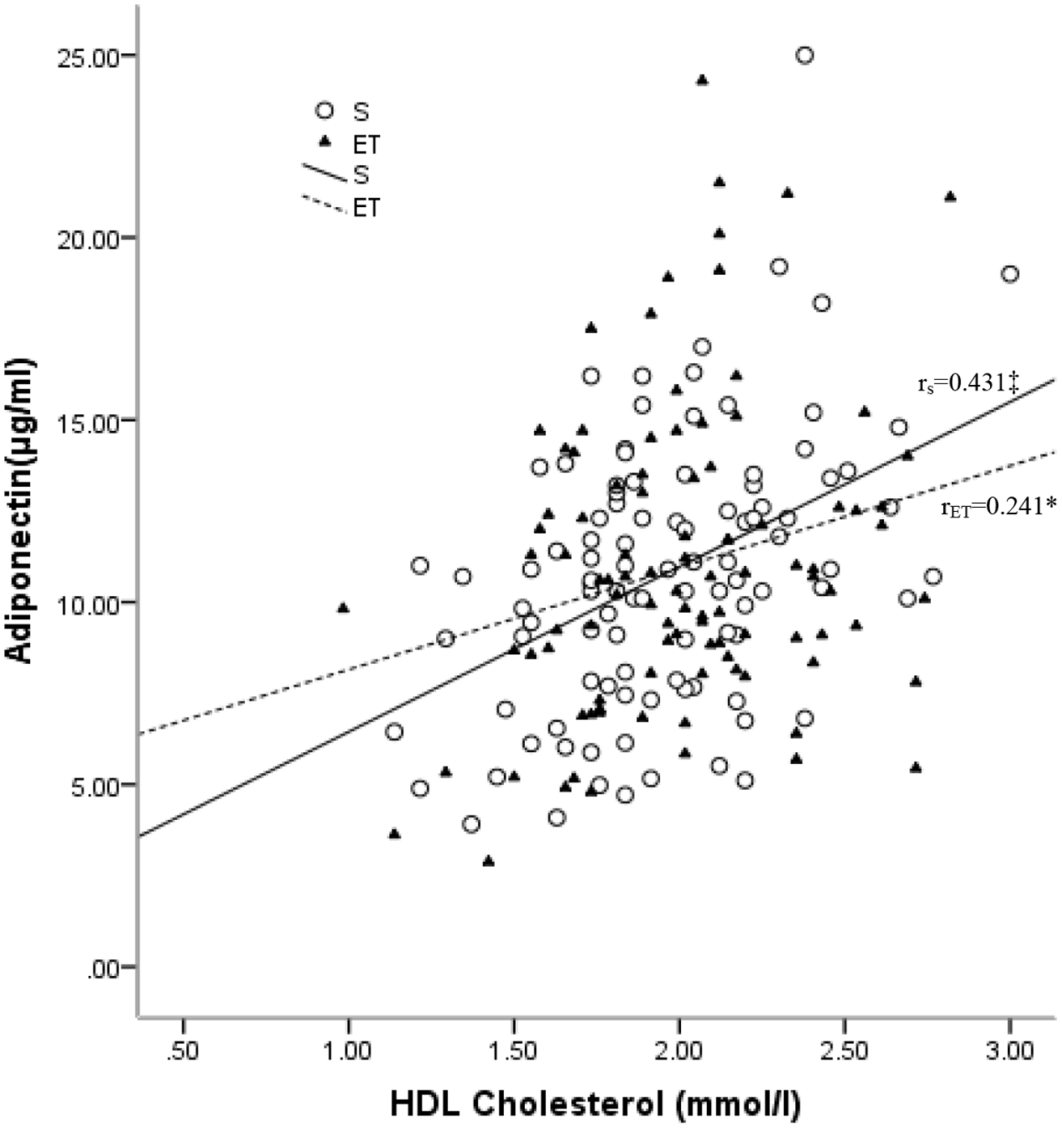
Relationships between plasma levels of adiponectin and HDL-C in the sedentary (S) and endurance-trained (ET) groups. R_S_: correlation coefficient of the sedentary group; r_ET_: correlation coefficient of the endurance-trained group. *: P < 0.05; †: P < 0.01; ‡: P < 0.001.

In the sedentary group, adiponectin was positively associated with HDL-C (r = 0.431, p < 0.001) and ApoA1(r = 0.373, p < 0.001) and reversely associated with TG (r = –0.297, p = 0.003) and ApoB/ApoA1 (r = –0.253, p = 0.011). After adjustment for TFM, positive associations with HDL-C (r = 0.420, p < 0.001), ApoA1 (r = 0.363, p < 0.001), and TC (r = 0.271, p = 0.007) were observed, as well as a reverse association with TG (r = –0.258, p = 0.01). In the endurance-trained group, positive associations of adiponectin with HDL-C (r = 0.241, p = 0.016, **Figure 3**) and ApoA1 (r = 0.223, p = 0.026) were observed. After adjustment for TFM, the positive associations remained significant (for HDL-C, r = 0.237, p = 0.018 and for ApoA1, r =0.218, p = 0.03). However, no significant association of adiponectin with LDL-C was observed in both groups (**Table 2**).

#### 3.2.3 Association of adiponectin with inflammatory markers

A reverse association of adiponectin with hsCRP (r = –0.196, p = 0.049) was found in the sedentary group but was not significant after the adjustment for TFM. In the endurance-trained group, a reverse association of adiponectin with PAI-1 (r = –0.216, p = 0.031) was observed, which remained significant (r = –0.210, p = 0.037) even after the adjustment for TFM (**Table 2**).

#### 3.2.4 Association of adiponectin with insulin resistance

No significant association was found between adiponectin and QUICKI in both groups before and after adjustment for TFM (**Table 2**).

#### 3.2.5 Association of LFM with serum lipids in the whole study cohort

After adjustment for TFM, LFM was positively associated with HDL-C (r = 0.160, p = 0.024) and negatively associated with ApoB/ApoA1(r = –0.144, p = 0.042). A borderline negative association with TG (r = –0.136, p = 0.055) was also observed in the whole study cohort. However, the associations failed to achieve significance after further adjustment for both TFM and adiponectin (**Table 3**).

**Table 3:** Partial correlations between LFM and serum lipids in the whole cohort.

	Adjustment for TFM		Adjustment for TFM and Adiponectin	
	r	P-Value	r	P-Value
TC	0.034	0.637	–0.043	0.549
TG	–0.136	0.055	–0.082	0.251
HDL-C	0.160	**0.024**	0.056	0.430
LDL-C	–0.031	0.66	–0.067	0.344
ApoA1	0.133	0.061	0.041	0.566
ApoB	–0.097	0.173	–0.103	0.146
ApoB/ApoA1	–0.144	**0.042**	–0.097	0.173

r, Partial correlation coefficient. Numbers in bold: with statistical significance.

### 3.3 Multivariate correlations

We performed multivariate linear regression analysis to determine the key predictors of adiponectin level among the variables that showed significant univariate associations with adiponectin. For the sedentary individuals, the L/Tr ratio and HDL-C were the strongest positive correlation factors, whereas TG was the negative correlation factor of adiponectin. HDL-C with L/Tr ratio and TG can explain the 29.5% variance of adiponectin in this study. In the endurance-trained group, the strongest predictors for adiponectin were the L/Tr ratio and HDL-C. The L/Tr ratio and HDL-C had a positive correlation with adiponectin. The two variables may jointly explain 13.5% of the variance of adiponectin in the model (**Table 4**).

**Table 4:** Multiple-regression analysis for adiponectin as a dependent variable.

Independent Variables	B	SE (B)	Standard B	P-Value
Sedentary (r^2 ^= 0.295)				
HDL-C	4.74	1.185	0.452	**<0.001**
L/Tr ratio	6.56	2.2	0.273	**0.004**
TG	–2.977	1.366	–0.221	**0.032**
Endurance-trained (r^2 ^= 0.135)				
L/Tr ratio	7.817	2.653	0.279	**0.004**
HDL-C	2.54	1.098	0.219	**0.023**

B, regression coefficient; SE(B), standard error of regression coefficient; Standard B, standard regression coefficient. Numbers in bold: with statistical significance.

## 4 Discussion

In this study, we unveiled that the endurance-trained young women were more sensitive to insulin compared with the sedentary women, but the two groups have similar adiponectin concentrations. In addition, the association between adiponectin and QUICKI did not reach significance, suggesting that adiponectin may not be involved in mediating the exercise-related improvement of insulin sensitivity. LFM was associated with a favorable lipid profile against CVDs in the whole cohort. However, this relationship disappeared after plasma adiponectin was taken into account, implying the involvement of adiponectin in the cardioprotective role of LFM. The HDL-C and L/Tr ratio had the strongest positive associations with adiponectin in both groups. The TG in the sedentary group and PAL-1 in the endurance-trained group were important factors that negatively correlated with adiponectin. These results suggest that the associations among adiponectin, lipid, and inflammatory factors vary in women with different physical activity statuses.

The negative relationships of adiponectin with BMI and total fat mass had been well documented (26, 27). Consistent with previous reports, the present research revealed a negative association of adiponectin with BMI and TFM in the sedentary Japanese women. However, no significant differences were found in the endurance-trained women. The most notable finding was that the total body fat mass, LFM, and L/Tr ratio were positively associated with adiponectin in both groups after adjustment for TFM. The positive association of total body fat mass with adiponectin may be a reflection of the correlation of LFM to adiponectin since it represents a major adipose deposition after adjustment for TFM. The multiple regression analysis revealed that the L/Tr ratio was the strongest predictor of adiponectin in both groups. Unlike other studies that emphasized the importance of abdominal fat mass, our observations suggested that LFM is an important determinant that is positively associated with adiponectin independent of TFM. One of the most important notions addressed in the current study was that LFM and TFM are likely to exert their impacts on circulating adiponectin levels in different ways. This hypothesis was supported by studies that found a lower adiponectin mRNA expression in the visceral adipose tissue compared with the subcutaneous adipose tissue, suggesting an antagonizing impact of intra-abdominal fat on adiponectin production (28). Another study in women with metabolic syndrome observed lower adiponectin mRNA expression levels in the visceral adipose tissue than the normal controls (29). A possible explanation for the difference in the production of adiponectin in different regional adiposities is that large visceral adipocytes with greater triglyceride storage produce less adiponectin than small adipocytes in the subcutaneous region (30). Given that large adipocytes are less insulin sensitive, the insulin sensitivity of adipocytes may be a determinant of adiponectin production (30).

In the current study, we found that LFM is associated with a favorable lipid profile against atherosclerosis. This observation is in line with our previous data (31) and other previous research (32), suggesting that the cardioprotective role of LFM is associated with an advantageous serum lipid-lipoprotein profile. However, these associations became non-significant after adiponectin was taken into account in the current study. These observations, together with the data implying that adiponectin gene mRNA expression is more abundant in the subcutaneous adipose tissue than in the visceral adipose tissue (28, 29), lead us to hypothesize that the antiatherogenic role of LFM may be mediated by adiponectin. Regarding the relationship between adiponectin and serum lipids, we found that plasma adiponectin is positively related to HDL-C and ApoA1 independent of TFM in both the sedentary and endurance-trained women. In addition, we found a negative association with TG exclusively in the sedentary subjects. These results suggest that adiponectin is associated with hepatic lipase (33) and exerts its lipid-modulating effect by antagonizing the activity of hepatic lipase, which hydrolyzes triglyceride and phospholipids in HDL particles (34). Moreover, adiponectin can reduce hepatic lipid accumulation by stimulating fat oxidation induced by AMP-activated protein kinase activation (35). A reduction of hepatic lipid content may, in turn, improve lipid catabolism in the liver (36). In our study, the endurance-trained subjects displayed lower LDL-C and ApoB levels than the sedentary women. This finding may be partially due to the fact that the endurance-trained women were more insulin-sensitive, resulting in an enhanced catabolic rate of triglyceride. Moreover, the endurance-trained women have less TFM deposition than the sedentary women, leading to a lesser supply of non-esterified fatty acids for synthesizing triglyceride in the liver. Since both groups had similar circulating plasma adiponectin concentrations, it is plausible that adiponectin plays different roles in lipid regulation in young women with different physical activities.

Concurrent with our previous report on a young, healthy Japanese male population (37) and another study carried out in Pima Indian children (38), a significant association between adiponectin and insulin resistance was absent in the present study. This may be due to the narrow range of the QUICKI index and the relatively low BMI levels of our study subjects. The relationship between adiponectin and insulin resistance has been shown to be adiposity-dependent. In a cross-sectional study comprising 1,196 adolescents, adiponectin was found to have a negative association with fasting insulin levels only in overweight and obese subjects, but this association was absent in lean adolescents (39). In addition, serum adiponectin levels have been shown to decrease parallel to weight gain, as well as the progression of insulin resistance, in rhesus monkeys (40). These findings suggest that adiponectin may contribute primarily to insulin action changes associated with adiposity change. Therefore, we predicted that the failure to demonstrate the independent relationship between adiponectin and insulin resistance assessed by QUICKI in young, healthy women suggests that adiponectin may be associated primarily with adiposity and then modified by insulin resistance.

This study has several potential limitations that should be further investigated. First, the study design was cross-sectional and had an observational nature, which does not imply causality. Second, the levels of high-molecular-weight isoforms of adiponectin were not assayed in this sample cohort; thus, the total adiponectin level may only be a surrogate of the analysis. Finally, the cohort was relatively homogenous with a small range of insulin resistance index; thus, the relationship between adiponectin and insulin resistance may be underestimated. Although confounders such as obesity, age, sex, cigarette smoking, alcohol drinking, and drug administration were controlled, whether the results can be extended to more insulin-resistant subjects, such as an obese population, remains unknown.

## 5 Conclusions

Body fat distribution, especially the ratio of LFM to TFM, joined with HDL-C, are two important determinants of adiponectin in both sedentary and endurance-trained healthy young women. No significant difference regarding circulating adiponectin levels was observed between the two groups, which may partially be due to them having a similar HDL-C and L/Tr ratio. In addition, TG in the sedentary women and PAI-1 in the endurance-trained women are negatively associated with adiponectin. These results suggest that adiponectin plays different roles in lipid modulation and anti-inflammation in women with different physical activity statuses. Furthermore, LFM is associated with a favorable lipid profile in the whole study cohort, which became absent when adiponectin was taken into account, suggesting that adiponectin may be involved in this association.

## Data availability statement

The raw data supporting the conclusions of this article will be made available by the authors, without undue reservation.

## Ethics statement

The studies involving human participants were reviewed and approved by the ethnic committee of Mukogawa Women’s University. The patients/participants provided their written informed consent to participate in this study.

## Author contributions

YG - Conceptualization, methodology, writing - original draft. FZ - Investigation, data curation. JZ - Investigation, data curation. XN - Investigation and resources, data curation. LS - Investigation, data curation. YX - Investigation, funding acquisition, writing - review and editing. JH - Investigation, data curation. TK - Investigation, funding acquisition, writing - review and editing. BW - Supervision, writing - review and editing, funding acquisition. BW supervised the study, had full access to all data in the study, and takes responsibility for the integrity of the data and the accuracy of the data analysis. All authors contributed to the article and approved the submitted version.

## Glossary

**Table:**

% total fat	percentage of body fat mass (total body fat mass divided by body weight)
% TFM	percentage of trunk fat mass (trunk fat mass divided by body weight)
AFM	arm fat mass
A/Tr	arm fat mass divided by trunk fat mass
ApoA1	apolipoprotein A-1
ApoB	apolipoprotein B-100
BMI	body mass index
CRP	C-reactive protein
CV	coefficient of variation
CVD	cardiovascular disease
DBP	diastolic blood pressure
DXA	dual X-ray absorptiometry
ELISA	enzyme-link immunosorbent assay
ET	endurance-trained
FM	fat mass
FPG	fasting plasma glucose
HDL-C	high-density lipoprotein cholesterol
hsCRP	highly sensitive C-reactive protein
L/Tr	lower-body-fat mass divided by trunk fat mass
LDL-C	low-density lipoprotein cholesterol
LFM	lower-body-fat mass
QUICKI	quantitative insulin-sensitivity check index
MRI	magnetic resonance imaging
PAI-1	plasminogen activator inhibitor-1
S	sedentary
SBP	systolic blood pressure
TC	total cholesterol
TFM	trunk fat mass
TG	triglycerides
TNF-α	tumor necrosis factor-α
WBC	white blood cell count
